# Mastoid Disease, Differential Diagnosis, Examination; When We Should Operate

**Published:** 1901-12

**Authors:** F. Pierce Hoover

**Affiliations:** Jacksonville, Florida, Formerly Lecturer in Otology, New York Polyclinic, Assistant Surgeon Manhattan Eye and Ear Hospital, New York City


### MASTOID DISEASE, DIFFERENTIAL DIAGNOSIS, EXAMINATION; WHEN WE SHOULD OPERATE.

BY F. PIERCE HOOVER, M.D., JACKSONVILLE, FLORIDA,

Formerly Lecturer in Otology, New York Polyclinic, Assistant Surgeon Manhattan Eye and Ear Hospital, New York City.

The article I herewith present pertains to a subject frequently discussed and written about, although not so often seen in general practice. My idea when this, one of the most serious diseases, should be operated upon may be similar to that of many surgeons and aurists, but for those who have seen but few, if any, cases of the kind I trust the following will be of some use and benefit:

It has been my good fortune never to have lost but one patient after an operation for mastoiditis, and I have had two hundred and sixty-five cases. When ever I get a *well-defined* case of mastoiditis I *always* cut. A clean incision never does any harm of itself if antiseptically performed, and no matter whether I find pus or not, in a vast number of cases the tension is relieved and the patient, if there be no diseased bone, rapidly recovers; whereas, if let alone, the broken-down tissue will increase and the bone become diseased. Examination in the region of the mastoid process is absolutely necessary in any inflammation of the middle ear, especially in acute and chronic suppurations, because the inflammation may have extended from other regions to this part, and if so it is likely to produce marked changes by involving the external osseous wall, the periosteum or the integument, which it is *very* important to discover in time for treatment to be applied. We can ascertain by moderate pressure of the finger whether there is an inflammation of the periosteum or of the skin, any enlargement of the bone or whether any fluctuation is present; also by the same pressure we can ascertain if pressure upon the bone causes pain and to what extent, and if so the part of the bone the greatest pain is felt, and whether or not a fistulous opening exists. We must from time to time examine the lymphatic glands on the mastoid process as they are frequently swollen and infililated, especially in inflammations of the external meatus and of the middle ear, more especially so in purulent affections. A most favorable sign, generally considered, is where the inflammation decreases. Too much care and attention cannot be paid to this, as many times we have just cause to be hankful we did make our examination in a painstaking and careful manner, a something the specialist more particularly appreciates than the general practitioner, for these cases, as a rule, are referred to the specialist by physicians not doing work in that line. In mastoid operations one should be thoroughly acquainted with the anatomy of the part so as to understand the exact situation of the bone and its landmarks. The bone between the middle fossa and the tympanum is *very* thin; one can thus see how easy pus can break through the bone, remembering the relation of the tempero-sphenoidal lobe and the cerebellum to the petrous bone, producing inflammation of the meninges, thrombosis of the lateral sinus or cerebellum. We thus can realize why cerebral disease is the result of disease of the ear.

Mastoiditis may or may not be accompanied by the presence of pus. A patient with an acute mastoiditis will complain of great pain behind the ear, sometimes extending up to the parietal bone and down the neck, the glands in many instances becoming involved, much tenderness on pressure. An ear which has been discharging frequently stops when pain is felt, or there may never have been a running ear previous to mastoid disease. If allowed to go without treatment, as many do, we notice in our clinical experience, pus will form, and we will then be able to get flunctuation. We must always bear in mind a mastoid and cerebral abscess frequently coexist. *Ninety* per cent. of the last named lie in a circle of about one inch radius, the center being one inch and a quarter above and behind the meatus.

Suspect cerebral abscess when an old chronic suppurating ear stops discharging and there is a rise of temperature, dull pain over mastoid, nausea and vomiting, pulse frequent, diarrhea, etc. The pain is not always located at the site of the lesion.

The diagnosis of a meningeal abscess from a mastoid is the former likely develops within the first three or four days; while the latter seldom develops before the first week; also in a meningeal abscess there is usually dullness early, deepening into coma, delirium, photophobia, contraction of the pupils and marked rigidity of the cervical glands. If there is a thrombosis of the lateral sinus and pyemia, the internal jugular vein, as well as the lateral sinus, will be involved by the thrombus and will be hard and cord-like, and the veins of the face will be apt to be swollen, pressure over the sinus may be painful, fluctuation of the temperature, sweating and general prostration, rigors, and eventually suppuration in the joints with pyemic symptoms referable to the lungs and liver. If there is a tumor it is ordinarily slow in growth and frequently attended with local symptoms at an early stage, while abscess has a more rapid course. Choked disk is in most cases prominent in tumor and is generally double and much more frequent than in abscess; furthermore, abscess is very common in the tempero-sphenoidal lobe or in cerebellum from ear diseases, while a tumor is rarely seen in these localities. The temperature is not apt to rise in tumor, but if an abscess is present there will be rapid fluctuation, most likely prolonged *subnormal* temperature. If the patient be a syphilitic he is more inclined to have a tumor than an abscess. In acute otitis media, with involvement of the mastoid, the simple methods that we should use are, leeches, ice and Wilde's incision. An acute inflammation of the ear seldom causes cerebral symptoms. We should not hesitate an instant to operate if the following conditions exist, viz.: If drainage is prevented in suppurative cases of otitis, either acute or chronic, by granulations or narrowing of the auditory canal; when mastoid is normal and we cannot remove the pus or cholesteomata; if there are positive symptoms of meningitis or cerebritis; if to prevent blood-poisoning and to accomplish drainage; if for continued pain over the mastoid, whether accompanied or not by edema. Do not fancy when a patient complains of a pain over the mastoid and pain at the same time elsewhere over the head that there must necessarily be a mastoiditis; the diagnosis will likely be neuralgia, which is most commonly the result of exposure of some kind, getting the feet wet or sitting in a draught, etc. When a patient comes to the office or clinic with a swelling behind the ear it is not necessarily a mastoiditis, as the swelling may be the result of a bite of some insect which will cause great pain, discomfort, tenderness on pressure and possibly fever. Cold applications and painting with tincture of iodine over the part will in most instances be all the treatment required.

The poison from the bite of an insect can cause a mastoiditis but seldom does so. Injuries to the ear frequently cause a mastoiditis, as a slap on the ear which is so often given children by their teachers as well as parents. If there is persistent headache, vomiting or mental dullness *without* rise of temperature the mastoids should be opened. Many times an early operation saves doing a graver one later on. By using a gouge and chisel the mastoid, antrum and cells can be laid open and flushed out with an antiseptic solution. All necrosed or carious bone and all clots should be removed and free drainage. Thrombosis of the lateral sinus is a more frequent result of mastoid disease. Middle ear suppurations cause a majority of brain abscesses. Never, for an instant, neglect a running ear, particularly if there is *no* odor to the discharge, for it is *then* most dangerous; as has been proven, they produce the most frequent cerebral results. Inspissated pus found in the ear is much to be feared, having pathogenic micro-organisms. The *most* common cause of mastoid disease is chronic suppurative inflammation of the middle ear. Should roughness of the bone be found when operating scrape same away, and always follow up any sinus with a grooved director and freely open it up. The after treatment of an operation on the mastoid is rest in bed, watch the pulse and temperature, have the patient kept well nourished and bowels regular and good hygiene. The first dressing should be removed the second day after operation unless a hemorrhage require its earlier removal, after which wound should be cleansed every day by syringing it thoroughly with an antiseptic solution, then pack with iodoform gauze. Should any granulations appear curette them away with a fine wire curette. The following case I operated on at Manhattan Eye and Ear Hospital, February 11, 1896, the only one it had been my misfortune to lose; being such an interesting one I will again report it in detail: M. C., age 47, born in Germany, a clerk. About nine weeks previous to date of entry, February 11, he was first troubled with his head. At times the pain on the left side was so great he would cry out aloud in agony while busily waiting on customers; he was compelled to resign his position on this account. Thinking it was from his ear, a former patient of mine brought him to me at Manhattan Hospital for treatment. Upon examination I found great pain and tenderness over mastoid portion of temporal bone, marked fluctuation extending over the left parietal bone. On following day he entered the hospital, his head was shaved and a solution (10 per cent.) of cocain was injected in the region of the greatest swelling; an incision was then made and a large amount of pus evacuated. On examination with a probe an opening was found in the bone, and necrosis extended quite a distance around it. Wound was dressed with iodoform gauze and the patient put to bed. February 14th patient was etherized and the wound cleaned, the first incision was enlarged and the periosteum separated, all necrosed bone removed with a sharp spoon and chisel. A large bunch of granulations was found on the inner surface of the bone; these were removed with a light wire curette. While operating a hemorrhage of dark blood occurred, supposed to be from the inferior petrosal sinus. Wound packed lightly with iodoform gauze and bandaged.

February 15.—Bandage removed, found considerable discharge, sleptlittle during night; gave hypodermic Magendie's sol. gtts. vi. Temperature 101 4-10.

February 16.—Syringed bichloride sol. 1-1000 in wound; pus present. Repacked as before. Temperature 104 4-10, respiration 28, pulse 112.

February 17.—Not so much pus; slept well night before. Syringed hydrogen peroxide and then hydrogen bichlor. 1-1000 in wound; bandaged.

February 18.—Has no pain; maximum temperature 102 4-10, respiration 28, pulse 82.

February 19.—Slept well at night, no pain ; the wound dressed as usual. Temperature 104 4-10, respiration 30, pulse 116. Ordered quinine sulph. grs. v. t i d, whiskey dr. iii t i d, the patient perspired profusely.

February 20.—Slept well. Very little pus ; patient flighty. Treatment same as yesterday.

February 21.—Very flighty this a. m. Restless during night; dressed as before, no pus.

February 22.—Not so restless last night; slept most of the time. Pulse weak and occasionally intermittent. Has slight muscular twitching. Ordered potass. brom. grs. xx every four hours; whiskey dr. ii every two hours. Temperature taken every two hours. No pus.

February 23.—Has not had an action for two days; ordered an enema, no result. Patient slept little until 4 a.m.; somewhat quieter than previous night. Very flighty this a.m. until 10 ; has since then been in a stupor. 2 p.m. Respiration deep and rapid ; pulse weak and irregular. Responds well to stimulants. Muscular twitchings of the entire body. 8:30 p.m. Pulse very weak and irregular. Treatment the same.

February 24.—Did not sleep at all during night. Pulse gradually becoming weaker. At 7 p.m, patient died.

### POST-MORTEM EXAMINATION.

When skull cap was taken off found the dura mater and pia mater and all coverings of membrane of brain edematous, so much so that all the lobes were pressed down, the frontal especially.

When the dura mater was cut through over cerebellum a fluid that looked semi-purulent, not normal, was found. The lateral sinus extending back on that side to junction of superior longitudinal sinus was a purulent thrombus; this also extended into the superior petrosal sinus. The inferior petrosal sinus was free. Dura mater not wounded at all. Granulations were found on dura mater, also on bone below. No polypus found in the tympanic cavity. In making post-mortem, after opening the calvarium, a spontaneous separation of the parts in the neighborhood of the mastoid occurred, disclosing to view a drum membrane,

*perfect*, and the malleus and other ossicles. The specimen was placed among the collection of pathological specimens in the hospital and was of the finest of the kind ever seen.

111 West Forsyth Street.

